# Changes in the status of p53 affect drug sensitivity to thymidylate synthase (TS) inhibitors by altering TS levels

**DOI:** 10.1038/sj.bjc.6603639

**Published:** 2007-03-06

**Authors:** E Giovannetti, H H J Backus, D Wouters, C G Ferreira, V M M van Houten, R H Brakenhoff, M-F Poupon, A Azzarello, H M Pinedo, G J Peters

**Affiliations:** 1 Department of Medical Oncology, VU University Medical Center, Amsterdam, The Netherlands; 2 Otolaryngology/Head and Neck Surgery, VU University Medical Center, Amsterdam, The Netherlands; 3 Department of Molecular Oncology, Institute Curie, Paris, France; 4 Crucell Holland BV, Leiden, The Netherlands; 5 Department of Pharmacology and Chemotherapy, University of Pisa, Pisa, Italy; 6 Instituto Nacional de Cancer, Rio de Janeiro, Brazil

**Keywords:** thymidylate synthase, p53, colon, 5-FU and antifolates

## Abstract

Colorectal cancer (CRC) resistance to fluoropyrimidines and other inhibitors of thymidylate synthase (TS) is a serious clinical problem often associated with increased intracellular levels of TS. Since the tumour suppressor gene p53, which is mutated in 50% of CRC, regulates the expression of several genes, it may modulate TS activity, and changes in the status of p53 might be responsible for chemoresistance. Therefore, this study was aimed to investigate TS levels and sensitivity to TS inhibitors in wild-type (wt) and mutant (mt) p53 CRC cells, Lovo and WiDr, respectively, transfected with mt and wt p53. Lovo 175X2 cells (transfected with mt p53) were more resistant to 5-fluorouracil (5-FU; 2-fold), nolatrexed (3-fold), raltitrexed (3-fold) and pemetrexed (10-fold) in comparison with the wt p53 parental cells Lovo 92. Resistance was associated with an increase in TS protein expression and catalytic activity, which might be caused by the loss of the inhibitory effect on the activity of TS promoter or by the lack of TS mRNA degradation, as suggested by the reversal of TS expression to the levels of Lovo 92 cells by adding actinomycin. In contrast, Lovo li cells, characterized by functionally inactive p53, were 3-13-fold more sensitive to nolatrexed, raltitrexed and pemetrexed, and had a lower TS mRNA, protein expression and catalytic activity than Lovo 92. However, MDM-2 expression was significantly higher in Lovo li, while no significant differences were observed in Lovo 175X2 cells with respect to Lovo 92. Finally, mt p53 WiDr transfected with wt p53 were not significantly different from mt p53 WiDr cells with respect to sensitivity to TS inhibitors or TS levels. Altogether, these results indicate that changes in the status of p53, can differently alter sensitivity to TS inhibitors by affecting TS levels, depending on activity or cell line, and might explain the lack of clear correlation between mutations in p53 and clinical outcome after chemotherapy with TS inhibitors.

Thymidylate synthase (TS) is a key enzyme in the *de novo* synthesis of deoxythymidine-5′-monophosphate (dTMP; Carreras and Santi, 1995), an essential precursor for DNA replication. Therefore, one of the first rational approaches to pharmacological treatment of colorectal cancer (CRC) was based upon fluoropyrimidine inhibitors of this enzyme, such as 5-fluorouracil (5-FU) (Danenberg *et al*, 1999; Rose *et al*, 2002). A major mechanism of resistance to 5-FU is overexpression of TS (Peters *et al*, 2002). At transcriptional level, TS expression is activated by the transcription factor E2F1, which plays a key role in cell cycle progression (DeGregori *et al*, 1995), and appears to be inhibited by the tumour suppressor gene p53 (Lee *et al*, 1997). In addition, TS protein functions as an RNA binding protein repressing the translation of its own mRNA (Chu *et al*, 1991, 1993), as well as of that of the oncogene c-myc (Chu *et al*, 1995) and of p53 (Ju *et al*, 1999).

The relationship between p53 and TS may be altered by mutations affecting p53, which occur in approximately 50% of CRC (Hollstein *et al*, 1991; Soussi *et al*, 2006). Mutations in p53 have been related with resistance to chemotherapy (Lowe *et al*, 1993) and CRC cells with p53 mutated or disrupted through homologous recombination were less sensitive to 5-FU (O'Connor *et al*, 1997; Bunz *et al*, 1999). However, no clear survival benefit for wild-type (wt) p53 was observed in our previous study of 5-FU treated CRC patients (Van Triest *et al*, 2000), and the presence of p53 mutations failed to predict which patients would benefit from 5-FU-based adjuvant chemotherapy in large retrospective clinical trials (Allegra *et al*, 2003; Russo *et al*, 2005). Indeed, although p53 is one of the most widely studied genes in CRC, yet there is no single guideline in gastrointestinal oncology recommending the routine analysis of p53 status for the assessment of drug response (Hoff, 2005).

In the present study, we aimed to clarify the role of p53 as a possible resistance factor for TS inhibitors. For this purpose, we used mutant (mt) and wt p53 transfected cell lines to study the effect of p53 mutations on TS expression and activity levels as well as on sensitivity to TS inhibitors characterized by different mechanism of action. In particular, human CRC cell lines Lovo 92 and WiDr T and their transfected variants were exposed to novel antifolate TS inhibitors and 5-FU, whose active metabolite 5-fluoro-2′-deoxyuridine-5′-monophosphate (FdUMP) competes with dUMP, and forms a ternary complex with 5,10-methylene-tetrahydrofolate and TS. The new antifolate TS-inhibitors include GW1843 and the quinazoline folate analogue raltitrexed, which act as a direct and specific TS inhibitor. The water-soluble lipophilic nolatrexed is a direct inhibitor of TS, which does not have glutamate side chains and can enter the cell by passive diffusion. The multitargeted antifolate pemetrexed, inhibits a range of enzymes involved in folate metabolism, including TS, dihydrofolate reductase (DHFR), human monofunctional glycinamide ribonucleotide formyltransferase, and aminoimidazole carboxamide ribonucleotide formyltransferase (Rose *et al*, 2002; Shafran *et al*, 2005).

## MATERIALS AND METHODS

### Drugs and chemicals

The TS inhibitors used in this study were provided by the following persons/institutions: 5-FU by Sigma Chemicals Co. (St Louis, MO, USA), raltitrexed by Astra-Zeneca Pharmaceuticals (Macclesfield, UK), pemetrexed by Eli Lilly Inc. (Indianapolis, IN, USA), GW1843U89 by Glaxo/Wellcome Co. (Research Triangle Park, NYC, USA) and nolatrexed by Zarix, Limited (King of Prussia, PA, USA). RNAzol was purchased from Campro Scientific (Veenendaal, The Netherlands); Moloney Murine Leukemia Virus Reverse Transcriptase (M-MLV-RT) from Promega (Madison, WI, USA); deoxynucleotides (dNTPs), random hexamers and Taq polymerase from Pharmacia Biotech (Roosendaal, The Netherlands). Acrylamide:bis (29 : 1) was purchased from Amresco research (Solon, Ohio, USA); nitrocellulose membranes, ECL plus and hyperfilm ECL from Amersham Life Science (Bucks, USA) and bovine serum albumine (BSA) from Boehringer Mannheim (Germany). Primary TS (clone 31) and p53 (Ab-2) antibodies were obtained from Dr GW Aherne (Sutton, UK) and from Oncogene Research products (Cambridge, MA, USA), respectively. Horse radish peroxidase conjugated secondary antibodies were obtained from Amersham Life Science (Bucks, PA, USA). Unless otherwise specified, all other chemicals were of analytical grade and commercially available.

### Cell lines

The human colon carcinoma cell lines Lovo 92 and WiDr T and their transfected variants were generously provided by Dr Poupon (Pocard *et al*, 1996) and Prof. Takahashi (Tamura *et al*, 1995). Lovo 92 (wt p53) and WiDr T (mt p53) are parental cell lines, and Lovo B2 and WiDr P empty vector plasmid controls. WiDr B is a cell line derived from WiDr T transfected with wt p53. Lovo 175X2 (mutation at position 175 (Arg → His)) cells are Lovo 92 cells transfected with mt p53. Lovo li is derived from Lovo 92 with functional inactive p53 but without p53 mutations. Functional activity of Lovo li was determined as described previously (Flaman *et al*, 1995). Briefly, p53 mRNA of Lovo li is reverse-transcribed, amplified by PCR, and co-transformed into yeast with an ADE2 open-reading frame under the control of a p53-responsive element. Yeast strains with functionally active p53 express ADE2 and form white colonies, whereas those with inactive p53 fail to express ADE2. Consequently, they form red colonies. The yeast strain transformed with p53 mRNA from Lovo 92 formed 15% of red colonies and those of Lovo li, 40%. Identification of p53 mutations was performed after sequencing of p53 exons 2–9 and exon 11. Therefore, all exons were separately amplified using PCR (primers available on request) as described previously by Sidransky *et al* (1991) and modified by Van Houten *et al* (2000).

All cell lines were cultured at 37°C in a 5% CO_2_ humidified atmosphere in RPMI (Flow Laboratories, Irvine, Scotland) supplemented with 10% FCS (GIBCO, Paisley, UK). Medium of the transfected cell lines was supplemented for selection with G418 (500 *μ*g ml^−1^). All cell lines were growing exponentially as monolayers during the course of all experiments. Cells used for determination of TS expression by PCR, for TS protein expression by western blotting and for TS catalytic and FdUMP binding assays were harvested, frozen as cell pellets in liquid nitrogen and stored at −80°C until use.

### Growth inhibition experiments

To evaluate the anti-proliferative effects of 5-FU, pemetrexed, raltitrexed, GW1843U89 and nolatrexed, we used an MTT (3-[4,5-dimethylthiazol-2-yl]-2,5-diphenyl tetrazolium bromide) assay, which produced similar results as a clonogenic assay (Ferreira *et al*, 2001). Lovo and WiDr cells (10 000 cells well^−1^) were exposed to various concentrations of TS inhibitors ranging from 10^−5^ to 10^−11^ M for 72 h. Thereafter, medium was removed and cells were incubated for 3 h at 37°C in 50 *μ*l MTT (final concentration: 0.42 mg ml^−1^). Formazan crystals were dissolved in 150 *μ*l of dimethylsulfoxide and the optical density was measured at 540 nm. IC_50_ values were defined as the concentrations that correspond to a reduction of cellular growth by 50% when compared to values of untreated control cells.

### Analysis of TS, DHFR and MDM-2, mRNA expression

RNA was extracted from 5 × 10^6^ cells by the RNAzol method and reversed transcribed by M-MLV-RT and random hexamers as described by the manufacturer. TS mRNA expression in all cell lines was studied by competitive template PCR (CT-PCR), using competitive templates designed for *β*-actin and TS dissolved in standardized dilutions (10^−12^/10^−14^, 10^−13^/10^−15^, 10^−14^/10^−16^ M, respectively). cDNA samples were amplified in a MJ Research PTC-2000 apparatus (Biozym, Landgraaf, the Netherlands) with 1 min steps of denaturation at 94°C, primer annealing at 58°C and elongation at 72°C for 35 cycles starting with a hot start at 94°C. PCR products were separated by 130 Volt electrophoresis for 2 h on 2% agarose gels containing 0.1 mg ml^−1^ ethidium bromide. The intensity of the native target (NT) and template bands was quantified by digital image analysis using Molecular Analist (Biorad, Veenendaal, the Netherlands). Concentrations of NT molecules of TS and *β*-actin in the cDNA samples were calculated by the ratio of NT/CT after amplification and the molarity of the CT mixture used, as described previously (Rots *et al*, 2000).

Additional analysis of TS and DHFR expression in the Lovo variants was performed by real-time PCR with the Applied Biosystems 7500HT sequence detection system (Applied Biosystems, Foster City, CA). Forward (F) and reverse (R) primers and probes (P) were designed with Primer Express 2.0 (Applied Biosystems) on the basis of TS and DHFR gene sequence obtained from the GeneBank. PCR reactions were performed in triplicate using 5 *μ*l of cDNA, 12.5 *μ*l of TaqMan Universal PCR Master Mix, 2.5 *μ*l of probe and 2.5 *μ*l of forward and reverse primers in a final volume of 25 *μ*l (Giovannetti *et al*, 2005).

Analysis of MDM-2 expression was performed by SYBR GREEN real-time PCR with the LightCycler Instrument (Roche Applied Science, Mannheim, Germany), as described previously (Slack *et al*, 2005).

Amplifications were normalized to *β*-actin, after that preliminary experiments carried out with dilutions of cDNA obtained from Quantitative PCR Human Reference Total RNA (Stratagene, La Jolla, CA, USA) demonstrated that the efficiencies of amplification of all targets and reference (*β*-actin) genes are approximately equal.

### Western blot analysis of TS and p53

Frozen pellets of Lovo and WiDr variants were lysed and 25 *μ*g total protein from each cell line was loaded and separated on a 10% SDS–PAGE gel (acrylamide:bis=29 : 1) followed by blotting onto nitrocellulose membrane. The nitrocellulose membrane was preincubated at room temperature in blocking buffer (0.5% milk powder, 0.5% BSA in TBS-T (10 mM Tris–HCl, pH 8.0, 0.15 M NaCl, 0.05% Tween-20)) to prevent aspecific antibody binding. After 1 h, the primary TS (1 : 500) and p53 (1 : 100) antibodies were added and incubated overnight at room temperature (RT). After 3 washing steps of 30 min in TBS-T, the blots were incubated for 1 h at RT with anti-rabbit (for TS) and anti-mouse (for p53) horse-radish peroxidase labelled secondary antibodies (Peters *et al*, 2000). Detection of antibody binding was measured using enhanced chemoluminescence (ECL plus). Densitometric analysis of the images captured on the VersaDoc 3000 instrument (Bio-Rad, Hertfordshire UK) was performed with the Kontron Analysis Image software (Kontron Electronik, Munich, Germany).

### FdUMP binding and TS catalytic activity

Cell pellets of Lovo and WiDr variants (20 × 10^6^ cells ml^−1^) were dissolved in an ice-cold 200 mM Tris-HCl buffer (pH 7.4) containing 20 mM *β*-mercaptoethanol, 100 mM NaF and 15 mM CMP. After sonification (3 × 5 s with intervals of 10 s) and centrifugation (14 000 g for 15 min at 4°C) the enzyme-containing suspension was split in several parts. Fifty *μ*l was used for determination of the protein content according to Bradford assay. The remaining volume was used for the FdUMP binding and TS catalytic assay previously described by Van Triest *et al* (1999).

### Evaluation of modulation of TS expression

To test whether the modulation of TS expression and activity could depend on different TS regulation, Lovo 92 and Lovo175X2 exponentially growing cells were incubated in 10% FCS-RPMI supplemented with the transcriptional inhibitor actinomycin (Sigma), used at a non-toxic concentration of 5 *μ*g ml^−1^. After 4 h, cells were harvested and used for analysis of TS expression by PCR and Western blot, as described above.

### Statistical analysis

Potential differences between the parental Lovo and WiDr cells and their transfectant variants for the various parameters were evaluated using the two-tailed unpaired Student's *t*-test. Changes were considered significantly different when *P*<0.05.

## RESULTS

### Sensitivity to TS inhibitors

To study a potential effect of p53 mutations on the sensitivity to TS inhibitors, we performed growth inhibition experiments in several previously transfected cells. However, as shown in Table 1, no significant differences in doubling times were observed between the Lovo and WiDr variants, varying from 34.5 to 46.5 h. For the empty vector plasmid control Lovo B2 and the parental cell line Lovo 92 no significant differences (*P*>0.05) were observed in sensitivity for 5-FU (1.9 *vs* 1.7 *μ*M), nolatrexed (4.6 *vs* 5.1 *μ*M), raltitrexed (21.3–30.7 nM), pemetrexed (325.0–417.0 nM) and GW1843 (2.4–5.5 nM), although Lovo B2 tended to be more sensitive to all the antifolates. Lovo 175X2 (transfected with mt p53) were more resistant compared to the wt p53 parental cell line Lovo 92 (*P*<0.05); 2-fold to 5-FU, 3-fold to nolatrexed, 3-fold to raltitrexed and 10-fold to pemetrexed. In contrast, the inactive status of p53 in Lovo li reduced IC_50_ values of nolatrexed, raltitrexed, pemetrexed and GW1843 by 3-fold, 5-fold, 4-fold and 13-fold, respectively, compared with the parental cell line.

WiDr T (mt p53) and WiDr B (transfected with wt p53) cells did not differ with respect to sensitivity to TS inhibitors (Table 1). Similar IC_50_ values of 5-FU (7.6–11.5 *μ*M), nolatrexed (1.8–2.7 *μ*M), raltitrexed (3.7–4.7 nM), pemetrexed (23.5–25.4 nM) and GW1843 (0.7–1.1 nM) were found in WiDr T, WiDr P and WiDr B (*P*>0.05).

### mRNA expression of TS, DHFR and MDM-2 in wt and mt p53 control and transfected cells

In order to determine whether p53 induced changes in TS and DHFR that would be responsible for differences in sensitivity we determined the mRNA expression of these factors potentially affecting efficacy of 5-FU and antifolates using both a CT and a real-time PCR.

**Figure 1:**
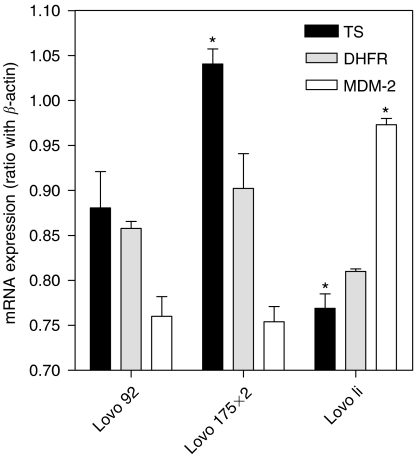
mRNA expression of TS, DHFR and MDM-2 in Lovo 92, Lovo 175X2 and Lovo li cells. The expression was determined by real-time PCR. Values were normalised to the simultaneously determined *β*-actin mRNA expression as described in the Material and Methods. Significant differences (*P*<0.05) between parental and transfected lines are indicated with ^*^.

The CT-PCR analysis showed that TS expression was 3-fold higher in Lovo 92 cells (average TS/*β*-actin ratio of 1.7 × 10^−2^), compared to WiDr T (0.3 × 10^−2^), while expression levels in all the WiDr variants were comparable (with values ranging from 0.3 to 0.5 × 10^−2^). Similarly, no significant differences were found between Lovo 92 and Lovo B2, while TS expression was 1.5-fold higher in Lovo 175X2 in comparison to Lovo 92 cells. In contrast, Lovo li cells were characterized by a significantly reduced mRNA expression of TS (0.4 × 10^−2^), compared to the parental cells. Real-time PCR analysis confirmed that Lovo li cells were characterized by a significantly reduced mRNA expression of TS compared to Lovo 92 cells (TS/*β*-actin ratio of 0.779±0.030 *vs* 0.880±0.032, *P*<0.05). Moreover, TS mRNA expression was significantly higher in Lovo 175X2 (1.041±0.024) compared to Lovo 92 cells (Figure 1). In contrast, no significant differences were observed in DHFR mRNA expression levels in Lovo 92, Lovo 175X2 and Lovo li cells (Figure 1).

Analysis of MDM-2 gene expression status revealed that Lovo li cells were characterized by significantly higher expression of MDM-2 mRNA with respect to Lovo 92 cells (MDM-2/*β*-actin ratio of 0.973±0.007 *vs* 0.760±0.022, *P*<0.05), while no significant differences were detected in Lovo 175X2 cells.

### Protein expression of TS and p53

**Figure 2:**
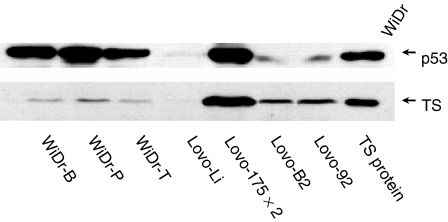
P53 and TS protein expression in p53 transfected colon cancer cell lines. Representative blot of at least three independent Western blotting analyses performed as described in the Material and Methods. WiDr cells (25 *μ*g total protein) and TS control protein (1 ng) were used as positive controls for the expression of p53 and TS, respectively. Western blot data has already been published (Backus *et al*, 2003).

Stable transfection of wt and mt p53 was checked by analysis of the p53 protein expression using western blot analysis (Figure 2). As expected, p53 expression was low in wt p53 cell lines Lovo 92 and Lovo B2, while up-regulation was found in the mt p53 transfectants Lovo 175X2. Hardly any p53 expression was detected in the p53 inactive cell line Lovo li. WiDr variants expressed high levels of p53 and WiDr B presented a higher expression of p53 than WiDr T, as detected by densitometric analysis. Western blotting also illustrated that the p53 status was associated with changes in the protein expression of TS. Low TS expression was detected in Lovo 92 and Lovo B2 cells, whereas an increase was found in Lovo 175X2. No TS expression was detectable in Lovo li. Low TS expression was found in WiDr T, WiDr P and WiDr B.

### FdUMP binding and TS catalytic activity

**Figure 3:**
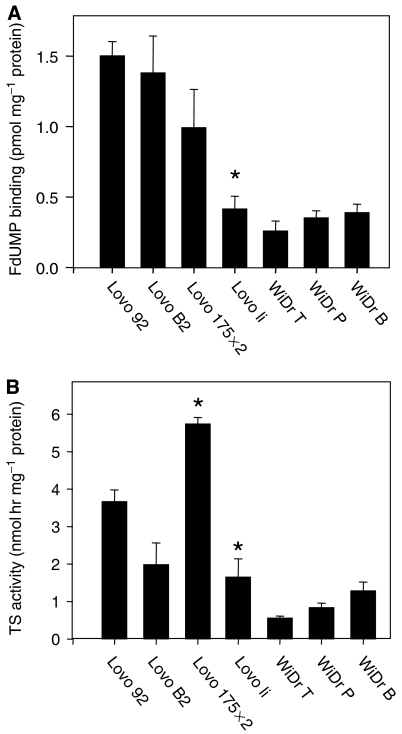
Changes in FdUMP binding and TS catalytic activity after transfection of mt p53 in colon cancer cells. Cell extracts were used for determination of (**A**) FdUMP binding (pmol mg^−1^ protein) and (**B**) TS activity (nmol h mg^−1^ protein). Results are given as mean values±s.e.m. of at least three experiments. Significant differences (*P*<0.05) between parental and transfected lines are indicated with ^*^.

Changes in TS protein expression were accompanied with differences in the number of FdUMP binding sites (Figure 3A) and TS catalytic activity (Figure 3B). Lovo li had significantly less FdUMP binding sites compared with Lovo 92, Lovo B2 and Lovo 175X2 cells. In addition, the catalytic activity of TS was about 2-fold lower in Lovo li compared with the parent cell line, while TS activity was about 1.5 higher in Lovo 175X2 (*P*<0.05). The number of FdUMP binding sites was comparable in all WiDr variants, as well as the TS catalytic activity.

### Modulation of TS expression by inhibition of transcription

**Figure 4:**
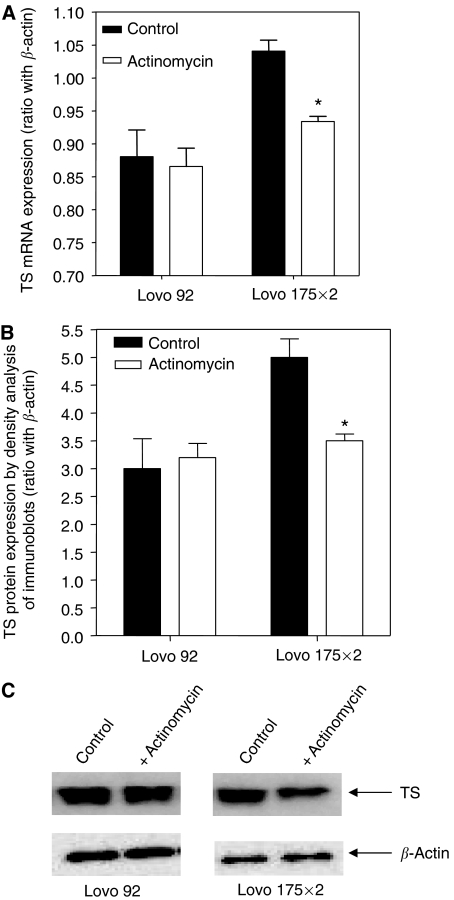
Changes in TS mRNA and protein expression after treatment with actinomycin in parental wt p53 (Lovo 92) and transfected mt p53 (Lovo 175X2) colon cancer cells. Analysis of (**A**) mRNA and (**B**) protein expression were performed with real time with quantitative PCR and Western blot, respectively. (**C**) Representative plot of Western blot analyses performed as described in the Material and Methods. Significant differences (*P*<0.05) between control and treated cells are indicated with ^*^.

To determine whether the increased TS expression in Lovo 175X2 cells was caused by modulation of transcriptional activity, cells were treated with actinomycin, using conditions widely employed to study inhibition of transcription. TS mRNA expression and protein expression were compared to that observed in the parental cell line Lovo 92. PCR analysis demonstrated that actinomycin significantly reduced mRNA TS expression in Lovo 175X2 cells, while a slight decrease of mRNA TS was observed in Lovo 92 cells (Figure 4A), suggesting that TS mRNA degrades at a faster rate in Lovo 175X2 cells as compared to Lovo 92 cells. These results were consistent with those of Figure 4B, showing that TS protein levels were minimally modulated in Lovo 92 cells, whereas TS levels were significantly reduced by actinomycin in Lovo 175X2 cells.

## DISCUSSION

This study demonstrates that changes in the status of p53 due to mutations or inactivity can alter sensitivity to TS inhibitors such as 5-FU and antifolates. Sensitive p53 inactive Lovo cells had low levels of TS whereas high TS levels were found in resistant mt p53 cells. However, transfection of wt p53 in mt p53 cells had no effect on TS levels and hence, also on sensitivity.

Mutations in the tumour suppressor gene p53 have been related with chemoresistance (Lowe *et al*, 1993) and mt p53 cells in the NCI cell line panel were less sensitive to 5-FU (O'Connor *et al*, 1997). In addition, in a panel of 14 colon cancer cell lines we observed that cells with a p53 mutation were more resistant to 5-FU and raltitrexed (Peters *et al*, 2002). In the present study, Lovo cells transfection with mt p53 increased TS mRNA and protein expression and activity which were associated with a decreased sensitivity to 5-FU and antifolates compared to the wt parental cell line Lovo 92. The increase in TS levels might be caused by the loss of the inhibitory effect of wt p53 on the promoter activity of TS as demonstrated by Lee *et al* (1997) suggesting that in Lovo 175X2 cells mt p53 interferes with the function of wt p53 in a dominant negative fashion (Fearon and Vogelstein, 1990). In the present study, the addition of the transcriptional inhibitor actinomycin in Lovo 175X2 cells caused a significant greater reduction of TS expression with respect to Lovo 92 cells, suggesting the occurrence of different rates of TS mRNA degradation.

On the other hand, sensitivity to 5-FU and antifolates in the CRC cells used in this study was not correlated with DHFR mRNA expression, whose levels were unchanged in Lovo 92, Lovo 175X2 and Lovo li cells. Similarly, resistance to the antifolates is probably not caused by the reduced folate carrier (RFC) and folylpolyglutamate synthetase (FPGS), both important for the activity of raltitrexed and pemetrexed (Peters and Jansen, 1996), because for nolatrexed, which is a RFC and FPGS independent specific TS inhibitor, IC_50_ values were also increased. In contrast to the mt p53 Lovo 175X2, functionally inactive p53 in Lovo li increased sensitivity to TS inhibitors, which was associated with a decrease in TS mRNA, protein and activity levels. Because we did not find p53 mutations in the analysed exons of Lovo li, we can only hypothesize that other mechanisms may be involved in the modulation of p53 activity in these cells. The effect of p53 suppressor gene can be influenced by modifications in the gene itself, but also by post-transcriptional modifications such as phosphorylation and changes in physical conformation, or by interaction with other cellular proteins, such as MDM-2 oncogene protein (Hupp *et al*, 1992; Finlay, 1993) Therefore, we studied MDM-2 gene expression and we showed that Lovo li cells were characterized by significantly higher expression of MDM-2. Previous studies demonstrated that MDM-2 overexpression can block the p53-mediated transactivation, depriving p53 gene of antineoplastic activity (Lev Bar-Or *et al*, 2000), and the use of anti-sense MDM-2 had a co-operative growth-inhibitory effect with different classes of cytotoxic drugs in colon cancer cells (Tortora *et al*, 2000). However, Lovo 175X2 cells had MDM-2 expression values similar to Lovo 92 cells, and several studies demonstrated that oncoviral proteins, such as adenovirus El B protein and human papilloma virus E6 protein (Lane and Benchimol, 1990) as well as altered phosphorylation pattern (Satyamoorthy *et al*, 2000), nuclear exclusion and sequestration in the cytoplasm can also affect the normal function of p53 (Moll *et al*, 1992).

These results might also explain why immunohistochemistry of p53 is not always associated with survival benefit in colorectal cancer patients treated with 5-FU based chemotherapy (Van Triest *et al*, 2000), although the lack of relationship with clinical outcome may also be related to the discrepancy between p53 immunostaining and mutation analysis (Momand *et al*, 1992). Immunostaining for p53 is positive for up-regulated p53, but it may or may not reflect a mutated status of the protein (Lenz *et al*, 1998; Kressner *et al*, 1999).

A large number of important genetic determinants have been identified thus far in tumours but only a few of them (TS and dihydropyrimidine dehydrogenase) have been tested prospectively (Smorenburg *et al*, 2006). The prognostic value of specific tumour markers may have been limited up to now by the (1) the small number of patients screened and marked variability in analytical methodology (Van Triest *et al*, 2000) and lack of quality controls among different studies due to the difficulties in obtaining suitable tissue samples; and (2) the lack of prospective trials and studies primarily designed to detect specific correlations between gene abnormalities and drug activity (Peters *et al*, 2004). The tumour suppressor gene p53 represents a sound example of the problematic link between the choice of reference methodology and the determination of clinical utility. This gene may be analysed by different methods, including sequencing, fluorescence *in situ* hybridisation and immunohistochemistry, which may show different results, and, even for a single type of analysis, the specific methodological procedure and the interpretation criteria may be subjected to considerable variability (Hoff, 2005; Van Triest *et al*, 2000; Soussi *et al*, 2006). A large number of articles show a variable role of p53, possibly depending on tumour type and the type of treatment. It has been suggested that p53 gene mutation is a significant and independent predictor of poor prognosis in CRC (Russo *et al*, 2005), but we lack validation in prospective clinical trials as a marker for the assessment of prognosis in CRC patients (Hoff, 2005). Similarly, because of the lack of preclinical studies to evaluate correlations between gene abnormalities and the cytotoxic effect of the most commonly used anticancer drugs, there is no single guideline recommending the analysis of p53 status to predict response to specific drugs.

In this study we showed that mutations in p53 have differential effects on sensitivity to TS inhibitors, which may influence response and survival after chemotherapy with TS inhibitors. In addition, inactive p53 cells with low p53 expression also have an effect on TS and possibly on clinical outcome. However, in agreement with several studies which demonstrated that p53 mutants were refractory to reactivation to the wt p53 phenotype (Kern *et al*, 1992; Sigal and Rotter, 2000), transfection of wt p53 in the mt p53 cell line WiDr had no effect on sensitivity to TS inhibitors and TS activity, suggesting that the function of p53 could not be restored after transfection of wt p53 in mt p53 cells.

Therefore, these results indicate that changes in the status of p53 due to mutations or functional inactivity change the sensitivity to TS inhibitors by altering the expression and activity of TS. The increase and decrease in sensitivity to TS inhibitors *in vitro* might explain why no clear correlation was found in clinical studies between mutations in p53 and clinical outcome. In conclusion, p53 is an important tumour suppressor gene that deserves additional investigation as a marker of therapeutic activity in CRC, and results obtained in the present study suggest that analysis of the exact status of p53 (e.g. wt or mt and functionally active or not) could be useful to predict clinical outcome after chemotherapy with TS inhibitors.

## Figures and Tables

**Table 1:** IC_50_ values of TS inhibitors and doubling times in p53 colon cancer cell lines

	**P53 status**	**Doubling time (hrs)**	**5-FU (*μ*M)**	**Nolatrexed (*μ*M)**	**Raltitrexed (nM)**	**Pemetrexed (nM)**	**GW1843 (nM)**
Lovo 92	wt p53; parental	34.5±3.4	1.73±0.15	5.15±0.85	30.7±0.7	417.0±83.0	5.5±1.5
Lovo B2	Plasmid control	44.7±4.0	1.87±0.58	4.57±0.72	21.3±7.2	325.0±104.1	2.4±0.8
Lovo 175X2	Transfected with mt p53	46.5±5.5	3.25±0.25^*^	19.00±1.00^*^	100.0±1.0^*^	3912.0±138.9^*^	5.3±0.3
Lovo li	Functional inactive p53	41.7±4.2	2.10±0.40	2.10±0.10^*^	5.7±0.7^*^	72.5±12.5^*^	0.8±0.3
WiDr T	mt p53; parental	36.7±3.7	9.33±1.76	2.30±0.48	4.0±0.4	23.3±4.4	0.8±0.1
WiDr P	Plasmid control	42.0±2.9	11.50±3.05	1.80±0.10	4.7±1.2	23.5±1.5	1.1±0.2
WiDr B	Transfected with wt p53	41.4±3.9	7.00±2.00	2.70±0.51	3.7±0.4	25.5±4.1	0.7±0.2

Growth inhibition was determined as described in the Materials and Methods section. IC_50_ values are given as mean values (in nM or *μ*M)±standard error of the mean (s.e.m.) of at least three experiments. Significant differences (*P*<0.05) between parental lines (Lovo 92 and WiDr T) and transfected cell lines are indicated with ^*^. Nolatrexed data have already been published and are included for comparison purposes (Backus *et al*, 2003).
